# Ethylene causes transcriptomic changes in *Synechocystis* during phototaxis

**DOI:** 10.1002/pld3.48

**Published:** 2018-03-15

**Authors:** Randy F. Lacey, Cidney J. Allen, Arkadipta Bakshi, Brad M. Binder

**Affiliations:** ^1^ Department of Biochemistry & Cellular and Molecular Biology University of Tennessee Knoxville TN USA; ^2^ Genome Science and Technology Program University of Tennessee Knoxville TN USA; ^3^ Present address: Department of Botany University of Wisconsin Madison WI USA

**Keywords:** ethylene receptor, extracellular polymeric substances, RNA‐seq, *Synechocystis*

## Abstract

Ethylene is well known as a plant hormone, but its role in bacteria is poorly studied. We recently showed that *Synechocystis* sp. Strain PCC 6803 has a functional receptor for ethylene, ethylene response 1 (Etr1), that is involved in various processes such as phototaxis in response to directional light and biofilm formation. Here, we use RNA sequencing to examine the changes in gene transcripts caused by ethylene under phototaxis conditions. Over 500 gene transcripts across many functional categories, of approximately 3700 protein‐encoding genes, were altered by application of ethylene. In general, ethylene caused both up‐ and downregulation of genes within a functional category. However, the transcript levels of amino acid metabolism genes were mainly upregulated and cell envelope genes were mostly downregulated by ethylene. The changes in cell envelope genes correlate with our prior observation that ethylene affects cell surface properties to alter cell motility. Ethylene caused a twofold or more change in 62 transcripts with the largest category of upregulated genes annotated as transporters and the largest category of downregulated genes annotated as glycosyltransferases which sometimes are involved in changing the composition of sugars on the cell surface. Consistent with changes in cell envelope, glycosyltransferase, and transporter gene transcripts, application of ethylene altered the levels of specific sugar moieties on the surface of cells. Light signaling from Etr1 involves two proteins (Slr1213 and Slr1214) and a small, noncoding RNA,* carbon stress‐induced RNA1* (*csiR1*). Application of ethylene caused a rapid, but transient, decrease in the transcript levels of *etr1*,* slr1213*, and *slr1214* and a rapid and prolonged decrease in *csiR1* transcript. Deletion of Slr1214 caused a large increase in *csiR1* transcript levels and ethylene lowered *csiR1* transcript. These data combined with prior reports indicate that ethylene functions as a signal to affect a variety of processes altering the physiology of *Synechocystis* cells.

## 1 INTRODUCTION

Ethylene is a gas that acts as a plant hormone to affect growth, development, and responses to stress (Abeles, Morgan, & Saltveit, 1992; Mattoo & Suttle, 1991). The first step in the perception of ethylene by plants is mediated by a family of receptors which contain a conserved ethylene‐binding domain at the N terminus (Schaller & Bleecker, 1995; Wang et al., 2006). These receptors are homologous to bacterial two‐component receptors and contain a histidine kinase‐like domain (Chang, Kwok, Bleecker, & Meyerowitz, 1993). In bacteria, two‐component receptors signal by autophosphorylation on a histidine residue, followed by transfer of the phosphate to an aspartate residue on a response regulator protein which often regulates transcription (Schaller, Shiu, & Armitage, 2011). It has been proposed that plants gained ethylene receptors and other two‐component‐like receptors, from the cyanobacterium that gave rise to chloroplasts (Kehoe & Grossman, 1996; Martin et al., 2002; Mount & Chang, 2002; Schaller et al., 2011; Timmis, Ayliffe, Huang, & Martin, 2004).

Many cyanobacteria bind ethylene and contain predicted ethylene‐binding proteins (Lacey & Binder, 2016; Rodriguez et al., 1999; Wang et al., 2006). We recently demonstrated that the cyanobacterium *Synechocystis* sp. Strain PCC 6803 (referred to here as *Synechocystis*) contains a functional ethylene receptor encoded by the *slr1212* gene locus, hereafter referred to as Etr1 for ethylene response 1 as originally designated by Ulijasz and others (Ulijasz et al., 2009). This receptor, also called positive phototaxis A (PixA) and UV intensity response Sensor (UirS), contains an ethylene‐binding domain at the N terminus, followed by a phytochrome‐like domain that responds to photons, and a C‐terminal histidine kinase domain (Lacey & Binder, 2016; Narikawa et al., 2011; Song et al., 2011; Ulijasz et al., 2009). Thus, this receptor has two distinct inputs regulating *Synechocystis* physiology, light and ethylene. This is one of several photoreceptors in *Synechocystis*, but is the only one that is predicted to respond to two signals (Schuergers, Mullineaux, & Wilde, 2017). Our prior research indicated that ethylene causes an increase in the number of type IV pili, an alteration in the transcript levels of several genes related to the formation or export of extracellular polymeric substances (EPS), and an increase in photosystem II levels; these changes required Etr1 (Lacey & Binder, 2016). Ethylene‐induced changes on the cell surface are linked to an increase in positive phototaxis in response to directional light and biofilm formation in nondirectional light (Lacey & Binder, 2016). Interestingly, *Synechocystis* cells bioengineered to overproduce ethylene also show an enhanced phototaxis response (Kuchmina et al., 2017).

Previous research has resulted in models of signal transduction from Etr1 in response to light (Narikawa et al., 2011; Ramakrishnan & Tabor, 2016; Song et al., 2011). In these models, it has been proposed that absorption of a photon by the phytochrome‐like domain of Etr1 results in activation of the histidine kinase domain and autophosphorylation on a histidine residue. This is followed by phosphotransfer to a conserved aspartate residue on Slr1213, a protein predicted to be a response regulator that functions as a transcription factor. Phosphotransfer to Slr1213 causes an increase in the transcription of a small RNA, *carbon stress‐induced RNA1* (*csiR1*) which is located between *slr1213* and *slr1214* in the genome (Ramakrishnan & Tabor, 2016). These changes are posited to cause changes in the activity of a second response regulator transcription factor, Slr1214, which appears to be required for signaling from this pathway (Lacey & Binder, 2016; Narikawa et al., 2011; Song et al., 2011). Interestingly, the Etr1 and Slr1214 proteins may physically interact (Sato et al., 2007), providing evidence that there is an alternate pathway that potentially involves phosphotransfer that bypasses Slr1213 or that there is feedback regulation from Slr1214. Ethylene signaling occurs independently of light signaling in Etr1, and we have proposed that ethylene acts antagonistically to photons (Lacey & Binder, 2016). However, the signaling that occurs in *Synechocystis* in response to ethylene is poorly understood.

Ethylene is biosynthesized by many microbes (Abeles et al., 1992), but *Synechocystis* sp. Strain PCC 6803 does not biosynthesize ethylene unless the precursor of ethylene biosynthesis in plants, methionine, is added (Guerrero, Carbonell, Cossu, Correddu, & Jones, 2012; Henry et al., 2017; May, 2001; Ungerer et al., 2012). However, even if this synthesis does not occur in nature, it is noteworthy that ethylene in the environment can come from other organisms that produce ethylene (Abeles et al., 1992) and light causes abiotic production of ethylene from organics dissolved in water (Ratte, Bujok, Spitzy, & Rudolph, 1998; Ratte, Plass‐Dülmer, Koppmann, Rudolph, & Denga, 1993; Wilson, Swinnerton, & Lamontagne, 1970). Indeed, abiotically produced ethylene affects plant growth (Buer, Masle, & Wasteneys, 2000; Buer, Wasteneys, & Masle, 2003). Thus, we hypothesize that ethylene functions as an environmental signal for *Synechocystis*. In support of this, ethylene concentrations in water can occur in the same range where we observe physiological effects (Swinnerton & Lamontagne, 1974; Swinnerton & Linnenborn, 1967). Thus, *Synechocystis* is likely to use ethylene as an environmental cue affecting various processes.

To explore this hypothesis, we used RNA sequencing (RNA‐seq) to examine global transcriptomic changes that occur in *Synechocystis* in response to ethylene during phototaxis. We found that long‐term application of ethylene caused a change in over 500 gene transcripts with a twofold or more upregulation of 17 gene transcripts and downregulation of 45 gene transcripts. Using quantitative real‐time RT–PCR, we determined that ethylene also causes a reduction in *csiR1* transcript levels. This downregulation required Etr1 and occurred within 30 minutes of application of ethylene. Ethylene downregulated transcript levels for many genes annotated as potentially being involved in the formation of EPS. Correlating with this, ethylene caused alterations in the binding of selected lectins to *Synechocystis* cells, showing that ethylene affects specific sugars in the EPS. We also observed a reduction in the transcript levels of two genes that encode for proteins involved in phycobilisome degradation which could relate to our prior results showing that photosystem II levels are increased with ethylene. These results suggest that ethylene causes global changes that affect *Synechocystis* physiology. Thus, ethylene may affect *Synechocystis* survival.

## 2 MATERIALS AND METHODS

### 2.1 *Synechocystis* strains and growth conditions

*Synechocystis* sp. Strain PCC 6803 cells were acquired from the Pasteur Institute and grown on 1% (w/v) agar with modified BG‐11 (Rippka, Deruelles, Waterbury, Herdman, & Stainier, 1979) containing 100 nM CuSO_4_ in continuous light (30 μmol m^−2^ s^−1^) at 24°C. For all analyses described below, colonies of cells were exposed to phototaxing conditions where they were treated with directional white light at a fluence rate of 30 μmol m^−2^ s^−1^ for 4 days at 28°C in the presence or absence of 1 μl/L ethylene (Lacey & Binder, 2016). Unless otherwise specified, experiments were conducted on wild‐type cells. In some cases, we examined cells where the *etr1* or *slr1214* gene was deleted (designated ΔEtr1 and ΔSlr1214, respectively). These mutants have previously been described (Lacey & Binder, 2016; Song et al., 2011).

### 2.2 RNA sequencing and analysis

Cells from phototaxing colonies were harvested and RNA was extracted as previously described (Lacey & Binder, 2016). Three biological replicates were used for each condition. RNA samples were sent to the Genome Sequencing and Analysis Facility at the University of Texas at Austin where sequencing was performed using the NextSeq 500 Illumina platform. This produced approximately 20 million single end 75 base pair reads per sample. All data analysis was conducted using the Sun Grid Engine on the Newton High Performance Computing cluster at the University of Tennessee‐Knoxville. The quality of the reads was analyzed using FastQC (Babraham Bioinformatics, Cambridge, UK). The reference *Synechocystis* sp. Strain PCC 6803 genome was downloaded along with the annotated genome file. Read trimming was performed with a 4:15 sliding window and a minimum read length of 75. The quality of the trimmed reads was then analyzed using FastQC and then indexed and mapped to the reference genome using Bowtie2 and Tophat (Langmead & Salzberg, 2012; Langmead, Trapnell, Pop, & Salzberg, 2009). Over 90% of the total reads were successfully mapped to the genome in each sample. The BAM files produced from Tophat were first sorted then converted to SAM format files. The mapped reads were then sorted and counted using SamTools and HTSeq (Anders, Pyl, & Huber, 2015). The read counts were loaded into R, and differential expression analysis was performed using DESeq2 (Love, Huber, & Anders, 2014). Principal component analysis of the data indicated minimal variance among the replicates in each condition. To account for false discovery rate, we used the Benjamini–Hochberg procedure and allowed a false discovery of 10% (*q *<* *0.05).

The RNA‐seq data discussed in this publication have been deposited in the NCBI's Gene Expression Omnibus (Edgar, Domrachev, & Lash, 2002) and are accessible through GEO Series Accession Number GSE93614 (https://www.ncbi.nlm.nih.gov/geo/query/acc.cgi?acc=GSE93614).

### 2.3 Lectin‐binding assay

To examine changes in carbohydrates in the EPS, we isolated cells from colonies of cells phototaxing in the presence or absence of ethylene and labeled the cells with fluorescein isothiocyanate (FITC)‐conjugated lectins obtained from Sigma‐Aldrich according to the methods of Wassim and others (Wasim, Bible, Xie, & Alexandre, 2009). We used concanavalin A (ConA) from *Canavalia ensiformis* to detect α‐D‐mannose and α‐D‐glucose, peanut agglutinin (PNA) from *Arachis hypogaea* to detect galactose(β1‐3)*N*‐acetyl‐*D*‐galactosamine, and *Ulex europaeus* agglutinin (UEA) to bind α‐L‐fucose and N,N’‐diacetylchitobiose. Aliquots were placed on a sterile 1% (w/v) agarose pad and observed with an Axio Observer Z1 microscope (Zeiss) using a 100 ×  objective and equipped with a GFP fluorescence filter. We obtained images of representative fields of view of cells from at least three colonies in each condition using identical exposure settings. The levels of fluorescence in 9 to 50 cells from each colony in each condition were quantified using ImageJ and the average fluorescence intensity determined for each replicate. For background measurements, we examined unlabeled cells under identical exposure settings. Data were normalized to the average background fluorescence intensity and represent the average ± *SD*.

### 2.4 Quantitative RT–PCR

Cells from phototaxing colonies were isolated, RNA was extracted, cDNA was synthesized, and qPCR was carried out as previously described (Lacey & Binder, 2016). Primers for qPCR were designed using the qPCR primer design software on the GenScript website. The primers used were as follows: 5′‐TACAGGTGTGGGATAACGGA‐3′ (forward) and 5′ CGCCACCGACATATTCATAG‐3′ (reverse) for *etr1*; 5′‐GAATGCCATTGGCTCAAA‐3′ (forward) and 5′‐CCTGCAAGAACTTGCCTAAA‐3′ (reverse) for *csiR1*; 5′‐AGCCAATCATCAACAGCAAC‐3′ (forward) and 5′‐ACGGTAATTCCTTGGTCGAG‐3′ (reverse) for *slr1213*; 5′‐AAAGTGGTTTGCATTGACGA‐3′ (forward) and 5′‐AAACGGCAAAGCTCATAACC‐3′ (reverse) for *slr1214*; 5′‐AGTATTTGCAAGATTATGGCCATA‐3′ (forward) and 5′‐CGTCCGGAGTAGAATTTCCAAAG‐3′ (reverse) for *sll5043*; and 5′‐GAGTTAACGATGGCTCGATCTGCTT‐3′ (forward) and 5′‐GGTAGGCAATAACAATTCCAAGC‐3′ (reverse) for *slr1452*. The *tryptophan synthase* gene (*trpA*) was used as an internal control and reference gene. Primers for *trpA* and *pilB1* have previously been described (Lacey & Binder, 2016). Transcript amounts were normalized to levels of *trpA* transcript (Zhang, Pendse, Phillips, Cotner, & Khodursky, 2008) using the method of Livak and Schmittgen (Livak & Schmittgen, 2001). We have previously shown that ethylene treatment has no significant effect on this gene (Lacey & Binder, 2016); a finding supported by the RNA‐seq data (Table S1). Data were then normalized to gene transcript levels observed in wild‐type cells maintained in ethylene‐free air.

## 3 RESULTS

### 3.1 Application of ethylene causes global transcript changes

**Figure 1:**
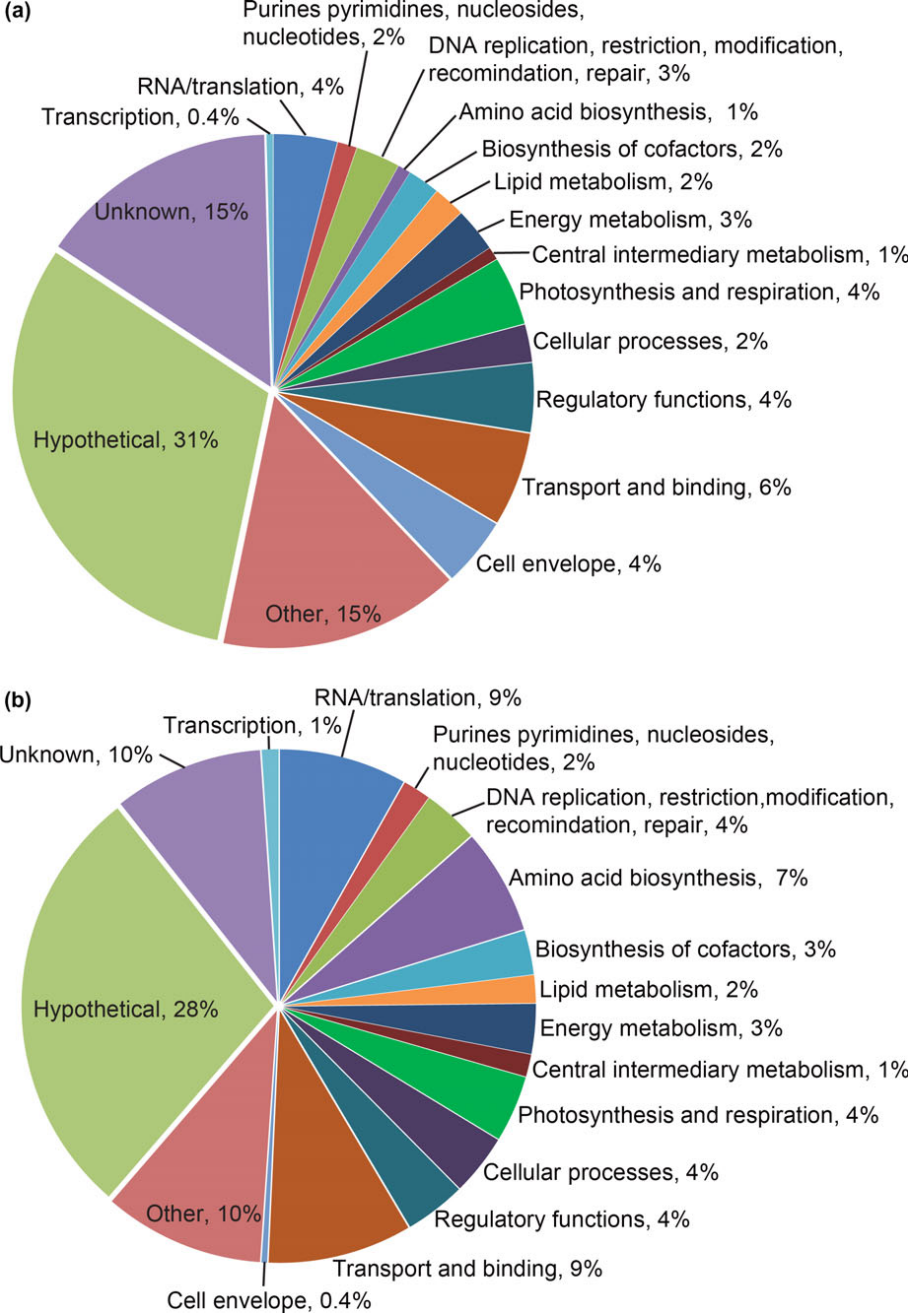
Pie chart showing the percent of gene transcripts altered by ethylene in various functional categories. The percent of total genes that were (a) downregulated or (b) upregulated by ethylene in each category is shown. Cells were maintained in phototaxis conditions for 4d in air or 1 μl/L ethylene. RNA‐seq analysis was carried out as described in the materials and methods, and differential gene expression was determined. Functional categories were then assigned for genes based on information from the CyanoBase website (http://genome.microbedb.jp/cyanobase/)

Application of ethylene to *Synechocystis* causes changes that affect motility, biofilm formation, and spontaneous cell sedimentation (Lacey & Binder, 2016). To understand the basis of these effects during phototaxis, we used RNA‐seq to examine global transcriptional changes that occur upon application of 1 μl/L ethylene. This concentration of ethylene has little or only a small effect on cell growth (Henry et al., 2017; Lacey & Binder, 2016) and is a standard concentration when studying ethylene signaling in plants. Cells were exposed to directional white light for 4 days in the absence or presence of ethylene, and then, RNA‐seq analysis was conducted as described in the materials and methods. We chose this long treatment protocol because the physiological changes we have previously observed in phototaxis, as well as biofilm formation and spontaneous cell sedimentation, were assessed after 3 to 4 days of ethylene treatment (Lacey & Binder, 2016). We mainly focused on the steady‐state changes that are linked to changes in phototaxis. Application of ethylene to *Synechocystis* caused changes in the transcript abundance of 533 annotated genes (of approximately 3700 protein‐encoding genes) with 282 upregulated and 251 downregulated using a false discovery rate < 0.05 (Figure S1, Table S1). An evaluation of the functions of these 533 annotated genes indicates that ethylene affects many processes including DNA and RNA biology, energy metabolism, metabolism of lipids and amino acids, photosynthesis, respiration, and transport across membranes (Figure 1). In general, ethylene caused a fairly equal up‐ and downregulation of gene transcripts within each category. The exceptions to this were that ethylene predominantly downregulated the transcript abundance of cell envelope‐related genes (11 of 12 genes) and upregulated transcript levels of amino acid biosynthesis‐related genes (19 of 22 genes). Similar to our previous results using real‐time RT–qPCR (Lacey & Binder, 2016), the transcript abundance of *pilB1* and *pilC* increased in response to ethylene; several other pilin‐related genes were also affected by ethylene. The alterations in pilin and cell envelope genes correlate with our prior observation that the main effect of ethylene on phototaxis occurs on the cell surface (Lacey & Binder, 2016). These surface changes would also affect biofilm formation and spontaneous cell sedimentation which ethylene also affects. Of the 64 genes in the *other* category, it is noteworthy that almost one‐third encode for putative glycosyltransferases, most of which are downregulated by the application of ethylene. Etr1 is one of over 40 histidine kinases in *Synechocystis*. It is therefore also interesting to note that ethylene affected the transcript abundance of 11 other histidine kinases and several response regulators, indicating that ethylene is likely to be affecting other signaling pathways.

Using a threshold of a twofold change, we determined that of these 533 annotated gene transcripts, ethylene caused the upregulation of 17 gene transcripts (Table 1) and downregulation of 45 gene transcripts (Table 2). Of the gene transcripts that were upregulated twofold or more, several are annotated as either transporters (*slr0753*,* slr0559*,* sll1598*,* sll1599*,* sll1600*) or transcriptional regulators (*slr0895*,* sll1392*) with the remainder being annotated for other processes. By contrast, the largest groups of downregulated gene transcripts with annotation are glycosyltransferases (*sll5043*,* sll5057*,* slr0820*,* slr1065*,* slr1070*,* slr1073*,* slr1076*,* slr2126*,* slr5054*,* slr5055*,* slr5056*) and methyltransferases (*sll1693*,* slr1068*,* slr1069*,* slr1071*,* slr1436*,* slr1610*). Using the Kyoto Encyclopedia of Genes and Genomes website (KEGG; http://www.genome.jp/kegg/), we evaluated whether or not these 62 genes encode for proteins involved in predicted pathways or complexes. Several proteins were revealed by this analysis to possibly work in complexes or pathways including Slr0559 that is predicted to work in complex with several genes not altered by ethylene (Slr0146, Slr0949, Slr0467, Slr1881) to transport amino acids, Sll1598 that interacts with Sll11599 and Sll1600 (both also altered by ethylene) in response to manganese starvation, and Slr1452 that interacts with Slr1453 (also altered by ethylene), Slr1454, and Slr1455 to regulate sulfur uptake.

**Table 1:** *Synechocystis* gene transcripts upregulated twofold or more by 1 μl/L ethylene

Gene locus number	Gene symbol (s)^a^	Description	Fold change	False discovery rate^b^	Replicon
*slr0895*	*prqR*	Transcriptional regulator	4.26	1.19 × 10^−15^	Chromosome
*slr0753*		Chloride transporter	3.07	1.42 × 10^−13^	Chromosome
*sll1600*	*mntB*	Manganese transporter	2.81	1.92 × 10^−4^	Chromosome
*sll0450*	*norB*	Cytochrome b subunit of nitric oxide reductase	2.77	2.85 × 10^−12^	Chromosome
*ssl1378*		Hypothetical protein	2.70	3.16 × 10^−11^	Chromosome
*sll0451*		Acetyltransferase	2.68	3.35 × 10^−14^	Chromosome
*slr0552*		Hypothetical protein	2.35	8.70 × 10^−10^	Chromosome
*sll0249*		Hydrolase	2.33	1.28 × 10^−2^	Chromosome
*sll0248*	*fldA, isiB*	Flavodoxin	2.23	1.63 × 10^−2^	Chromosome
*sll1599*	*mntA*	Manganese transporter	2.19	3.48 × 10^−2^	Chromosome
*sll1598*	*mntC*	Manganese transporter	2.16	4.19 × 10^−2^	Chromosome
*slr0551*		Ribonuclease J homolog	2.14	1.70 × 10^−8^	Chromosome
*sll1392*	*pfsR*	Transcriptional regulator	2.11	1.68 × 10^−3^	Chromosome
*slr7041*		mRNA interferase	2.05	5.77 × 10^−4^	pSYSA
*ssl0461*		Hypothetical protein	2.05	3.14 × 10^−2^	Chromosome
*ssr1038*		Unknown protein	2.01	1.17 × 10^−9^	Chromosome
*slr0559*	*natB*	Periplasmic binding protein of ABC transporter for natural amino acids	2.01	2.83 × 10^−7^	Chromosome

a Gene symbols and annotations from the CyanoBase, Cyanobacteria Gene Annotation Database, and Kyoto Encyclopedia of Genes and Genomes websites.

b A transcript was considered to be altered with a twofold or higher change with a false discovery rate < 0.05.

**Table 2:** *Synechocystis* gene transcripts downregulated twofold or more by 1 μl/L ethylene

Gene locus number	Gene symbol (s)^a^	Description	Fold change	False discovery rate^b^	Replicon
*slr1452*	*sbpA*	Sulfate transport system substrate binding protein	−5.99	5.22 × 10^−14^	Chromosome
*sll5043*		Glycosyltransferase	−5.11	4.27 × 10^−8^	pSYM
*slr5054*		Glycosyltransferase	−4.28	3.22 × 10^−12^	pSYM
*sll5044*		Unknown protein	−4.19	5.56 × 10^−7^	pSYM
*slr0269*		Hypothetical protein	−3.87	6.93 × 10^−10^	Chromosome
*ssr2439*		Hypothetical protein	−3.83	2.00 × 10^−6^	Chromosome
*slr5056*		Glycosyltransferase	−3.44	1.89 × 10^−6^	pSYM
*slr6031*		Unknown protein	−3.26	6.02 × 10^−4^	pSYSX
*slr2126*		Glycosyltransferase	−3.20	4.98 × 10^−10^	Chromosome
*sll5041*		Putative transposase	−2.94	3.16 × 10^−9^	pSYM
*slr5053*		Unknown protein	−2.85	1.75 × 10^−4^	pSYM
*sll5046*		Unknown protein	−2.81	1.68 × 10^−4^	pSYM
*slr0820*	*gumD*	Glycosyltransferase	−2.78	1.11 × 10^−5^	Chromosome
*slr1457*	*chrA*	Chromate transporter	−2.77	1.17 × 10^−3^	Chromosome
*slr0270*		Hypothetical protein	−2.68	2.10 × 10^−4^	Chromosome
*sll1696*		Hypothetical protein	−2.65	1.97 × 10^−4^	Chromosome
*ssl0453*	*nblA2, nblA*	Phycobilisome degradation	−2.59	4.69 × 10^−4^	Chromosome
*ssl0452*	*nblA1, nblA*	Phycobilisome degradation	−2.55	4.80 × 10^−4^	Chromosome
*sll1693*		Methyltransferase	−2.53	3.47 × 10^−6^	Chromosome
*slr2125*		Hypothetical protein	−2.48	6.28 × 10^−6^	Chromosome
*ssr2016*		Required for ferredoxin:plastoquinone reductase	−2.45	6.58 × 10^−3^	Chromosome
*sll0218*		Hypothetical protein	−2.42	1.88 × 10^−3^	Chromosome
*ssl3044*		Ferredoxin	−2.41	1.89 × 10^−6^	Chromosome
*sll5042*		Sulfotransferase	−2.35	1.45 × 10^−3^	pSYM
*slr1069*		Methyltransferase	−2.33	3.16 × 10^−11^	Chromosome
*slr5055*		Mannosaminuronic acid transferase	−2.30	2.24 × 10^−3^	pSYM
*sll0082*		Hypothetical protein	−2.27	1.99 × 10^−3^	Chromosome
*slr1704*		Hypothetical protein	−2.22	1.79 × 10^−3^	Chromosome
*slr1068*		Methyltransferase	−2.22	1.62 × 10^−7^	Chromosome
*slr1074*		Unknown protein	−2.18	1.11 × 10^−7^	Chromosome
*sll5057*		Glycosyltransferase	−2.17	5.88 × 10^−3^	pSYM
*slr0362*		Hydrolase	−2.17	2.11 × 10^−6^	Chromosome
*slr1071*		Methyltransferase	−2.13	8.30 × 10^−10^	Chromosome
*slr1215*		Hypothetical protein	−2.13	2.83 × 10^−7^	Chromosome
*slr1076*		Glycosyltransferase	−2.12	2.23 × 10^−16^	Chromosome
*sll0808*	*ISY508a*	Putative transposase	−2.10	2.99 × 10^−3^	Chromosome
*slr1456*	*pilA4, gspG*	Type 4 pilin‐like protein or general secretion pathway protein G	−2.09	1.03 × 10^−2^	Chromosome
*slr1070*		Glycosyltransferase	−2.08	6.40 × 10^−8^	Chromosome
*slr1065*		Glycosyltransferase	−2.08	1.77 × 10^−7^	Chromosome
*slr0361*		rRNA pseudouridine synthase	−2.04	6.31 × 10^−6^	Chromosome
*slr1436*		Methyltransferase	−2.04	1.34 × 10^−3^	Chromosome
*sll1772*	*mutS*	DNA mismatch repair protein	−2.04	8.69 × 10^−7^	Chromosome
*slr1073*		Glycosyltransferase	−2.02	1.54 × 10^−8^	Chromosome
*slr1610*		Putative C‐3 methyl transferase	−2.02	7.24 × 10^−12^	Chromosome
*slr0079*	*gspE*,* pilB2*	General secretion pathway protein E, *pilB* homolog	−2.01	7.40 × 10^−3^	Chromosome

a Gene symbols and annotations from the CyanoBase, Cyanobacteria Gene Annotation Database, and Kyoto Encyclopedia of Genes and Genomes websites.

b A transcript was considered to be altered with a twofold or higher change with a false discovery rate < 0.05.

The change in so many glycosyltransferases, as well as other carbohydrate metabolism‐related genes, cell envelope genes, and transporters, is consistent with the idea that carbohydrate metabolism is altered by ethylene to affect EPS formation or export or both. In support of this, four of the affected genes (*slr0982*,* slr1875*,* sll5052*, and *slr1610*) have previously been shown to be functional in EPS formation to affect biofilm formation (Fisher, Allen, Luo, & Curtiss, 2013; Jittawuttipoka et al., 2013) and four are predicted to be functional for EPS formation based on phylogenetic comparisons (*slr1074*,* slr0896*,* sll1377*, and *slr5056*) (Pereira, Mota, Vieira, Vieira, & Tamagnini, 2015). Changes in EPS are predicted to affect motility and biofilm formation in *Synechocystis* (Schuergers et al., 2017). We also previously found that ethylene increases the level of photosystem II (Lacey & Binder, 2016). Two genes, *ssl0452* (*nblA1*) and *ssl0453* (*nblA2*), were downregulated over twofold and are involved in phycobilisome degradation, suggesting that ethylene may also increase the levels of the phycobilisome light harvesting complex.

An examination of the *Synechocystis* sp. Strain PCC 6803 gene map on the KEGG website indicated that many of these 62 genes form clusters on the genome with similar patterns of change when ethylene was added (Tables 3 and 4). The largest group of genes that appear coregulated includes eight genes that were downregulated (*slr1065*,* slr1068*,* slr1069*,* slr1070*,* slr1071*,* slr1073*,* slr1074*, and *slr1076*). Interestingly, other genes in this region of the genome include *slr1063*,* slr1064*,* slr1066*,* slr1067*,* slr1072, slr1077*, and *slr1078* which also were downregulated by ethylene, but less than the twofold cutoff (Table 4). Using the Database of Prokaryotic Operons (DOOR^2^; http://csbl.bmb.uga.edu/DOOR/) revealed that most of these groups are predicted to be in operons. The exceptions were several pairs of genes (*sll1392* and *sll1393*;* sll0361* and *sll0362*;* sll1063* and *sll1064*) and *sll5041* through *sll5046*. The group of 13 genes from *slr1065* to *slr1078* is predicted to be in three operons.

**Table 3:** Selected clusters of genes upregulated by ethylene^a^

Gene locus	Gene symbol (s)^b^	Description	Fold change	False discovery rate
*sll0248*	*fldA, isiB*	Flavodoxin	2.23	1.63 × 10^−2^
*sll0249*		Hydrolase	2.33	1.28 × 10^−2^
*sll0250* c	*dfp*	Pantothenate metabolism flavoprotein	1.77	5.22 × 10^−2^
*sll0450*	*norB*	Cytochrome b subunit of nitric oxide reductase	2.77	2.85 × 10^−12^
*sll0451*		Acetyltransferase	2.68	3.35 × 10^−14^
*sll1392*	*pfsR*	Transcriptional regulator	2.11	1.68 × 10^−3^
*sll1393*	*glgA*	Glycogen/starch synthase	1.19	4.06 × 10^−2^
*sll1598*	*mntC*	Manganese transporter	2.16	4.19 × 10^−2^
*sll1599*	*mntA*	Manganese transporter	2.19	3.48 × 10^−2^
*sll1600*	*mntB*	Manganese transporter	2.81	1.92 × 10^−4^
*slr0551*		Ribonuclease	2.14	1.70 × 10^−8^
*slr0552*		Hypothetical protein	2.35	8.70 × 10^−10^
*slr0553*		Unknown protein	1.71	3.40 × 10^−4^
*slr0895*	*prqR*	Transcriptional regulator	4.26	1.19 × 10^−15^
*slr0896*		Multidrug and toxin extrusion (MATE) family	1.52	5.25 × 10^−3^

a Gene transcripts that showed a twofold or more increase upon treatment with ethylene were grouped with surrounding genes that showed increases.

b Gene names and descriptions from the CyanoBase, Cyanobacteria Gene Annotation Database, and Kyoto Encyclopedia of Genes and Genomes websites.

c The change in this gene had a false discovery rate that was slightly higher than 0.05.

**Table 4:** Selected clusters of genes downregulated by ethylene^a^

Gene locus	Gene symbol (s)^b^	Description	Fold change	False discovery rate
*sll0217* c	*flv4*	Potential FMN protein	−1.25	5.18 × 10^−02^
*sll0218*		Hypothetical protein	−2.42	1.88 × 10^−03^
*sll0219*	*flv2*	Potential FMN protein	−1.71	5.74 × 10^−03^
*sll1693*		Methyltransferase	−2.53	3.47 × 10^−06^
*sll1694*	*hofG*	Type IV pilus assembly protein	−1.95	9.42 × 10^−03^
*sll1695*	*pilA2*	Type IV pilus protein	−1.82	1.91 × 10^−02^
*sll1696*		Hypothetical protein	−2.65	1.97 × 10^−04^
*sll1771*		Hypothetical Protein	−1.97	4.85 × 10^−05^
*sll1772*	*mutS*	DNA mismatch repair protein	−2.04	8.69 × 10^−07^
*sll5041*		Putative transposase	−2.94	3.16 × 10^−09^
*sll5042*		Sulfotransferase	−2.35	1.45 × 10^−03^
*sll5043*		Glycosyltransferase	−5.11	4.27 × 10^−08^
*sll5044*		Unknown protein	−4.19	5.56 × 10^−07^
*sll5046*		Unknown protein	−2.81	1.68 × 10^−04^
*slr0361*		rRNA pseudouridine synthase	−2.04	6.31 × 10^−06^
*slr0362*		hydrolase	−2.17	2.11 × 10^−06^
*slr1063*		Glycosyltransferase	−1.86	1.67 × 10^−06^
*slr1064*	*rfbU, mtfA*	Mannosyltransferase B	−1.85	3.36 × 10^−07^
*slr1065*		Glycosyltransferase	−2.08	1.77 × 10^−07^
*slr1066*		Glycosyltransferase	−1.91	5.60 × 10^−07^
*slr1067*	*galE*	UDP‐glucose‐4‐epimerase	−1.43	8.91 × 10^−03^
*slr1068*		Methyltransferase	−2.22	1.62 × 10^−07^
*slr1069*		Methyltransferase	−2.33	3.16 × 10^−11^
*slr1070*		Glycosyltransferase	−2.08	6.40 × 10^−08^
*slr1071*		Methyltransferase	−2.13	8.30 × 10^−10^
*slr1072*	*yefA*	GDP‐D‐mannose dehydratase	−1.81	6.49 × 10^−06^
*slr1073*		Glycosyltransferase	−2.02	1.54 × 10^−08^
*slr1074*		Unknown protein	−2.18	1.11 × 10^−07^
*slr1076*		Glycosyltransferase	−2.12	2.23 × 10^−16^
*slr1077*	*gumH*	Glycosyltransferase	−1.89	3.16 × 10^−11^
*slr1078*	*galE*	Galactose metabolism	−1.56	2.99 × 10^−05^
*slr1436*		Methyltransferase	−2.04	1.34 × 10^−03^
*slr1437*		Unknown protein	−1.52	2.14 × 10^−03^
*slr1438* c		Aldose epimerase	−1.24	5.63 × 10^−02^
*slr1452*	*sbpA*	Sulfate transport protein	−5.99	5.22 × 10^−14^
*slr1453*	*cysT*	Sulfate transport system permease	−1.66	2.10 × 10^−02^
*slr1456*	*pilA4, gspG*	Type 4 pilin‐like protein	−2.09	1.03 × 10^−02^
*slr1457*	*chrA*	Chromate transporter	−2.77	1.17 × 10^−03^
*slr2125*		Methyltransferase	−2.48	6.28 × 10^−06^
*slr2126*		Hypothetical protein	−3.20	4.98 × 10^−10^
*slr5053*		Unknown protein	−2.85	1.75 × 10^−04^
*slr5054*		Glycosyltransferase	−4.28	3.22 × 10^−12^
*slr5055*		Mannosaminuronic acid transferase	−2.30	2.24 × 10^−03^
*slr5056*		Glycosyltransferase	−3.44	1.89 × 10^−06^
*ssl0452*	*nblA1, nblA*	Phycobilisome degradation protein	−2.55	4.80 × 10^−04^
*ssl0453*	*nblA2, nblA*	Phycobilisome degradation protein	−2.59	4.69 × 10^−04^

a Gene transcripts that showed a twofold or more increase upon treatment with ethylene were grouped with surrounding genes that showed increases.

b Gene names and descriptions from the CyanoBase, Cyanobacteria Gene Annotation Database, and Kyoto Encyclopedia of Genes and Genomes websites.

c The change in this gene had a false discovery rate that was slightly higher than 0.05.

### 3.2 Real‐time RT–qPCR of several ethylene‐responsive genes

**Figure 2:**
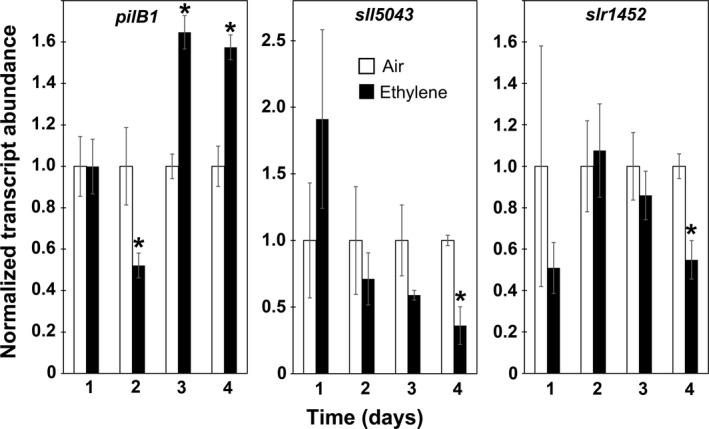
Real‐time RT–qPCR of Selected Ethylene‐Responsive Transcripts. Normalized transcript abundance of four genes shown to be affected by ethylene using RNA‐seq. Cells were maintained in phototaxis conditions for the designated times in the absence or presence of 1 μl/L ethylene. RNA was isolated, and real‐time RT–qPCR was conducted. Data were normalized to the *trpA* reference gene transcript and then normalized to levels in cells maintained in ethylene‐free air for that time‐point. Data represent the average ± *SEM* of two biological replicates with three technical replicates per biological replicate. * designates statistically significant difference (*p *<* *.05) caused by application of ethylene at that time‐point as determined by Student's *t* test

We wished to further characterize the transcriptional changes induced by application of ethylene. To do this, we used real‐time RT–qPCR to examine the transcript levels of three genes identified by the RNA‐seq analysis. Two of these were downregulated (*slr1452*,* sll5043*) and one upregulated (*pilB1*). The *slr1452* gene is annotated as involved in sulfate transport and the *sll5043* as a glycosyltransferase. The *pilB1* gene is involved in type IV pili function (Yoshihara et al., 2001). Using real‐time RT–qPCR, we previously showed that *pilB1* is upregulated by long‐term application of ethylene (Lacey & Binder, 2016), consistent with the RNA‐seq data; we obtained similar results in this study (Figure 2). We also confirmed that, consistent with RNA‐seq results, *slr1452* and *sll4043* are downregulated by 4d treatment with 1 μl/L ethylene (Figure 2). We were interested to know whether or not ethylene rapidly affects the transcript levels of these genes. We first tested for transcriptional changes within the first 4 hr of ethylene treatment. This revealed that there were fluctuations in the transcript levels of *slr5043* and *slr1452*, but ethylene caused no statistically significant changes in this time period (Figure S2). By contrast, *pilB1* transcript levels decreased by 30 min after starting the ethylene treatment. However, the levels of this gene also decreased at 240 min to the same extent whether or not ethylene was present, suggesting that ethylene may not be causing rapid changes in this gene. To further explore the time‐course of changes in these genes, we examined transcript changes at longer times of ethylene treatment. The transcript levels of *pilB1* were reduced by a two‐day application of ethylene (Figure 2). However, by day 3, ethylene caused an increase in *pilB1* transcript levels over air controls. By contrast, neither *sll5043* nor *slr1452* showed a statistically significant (*p *<* *.05) response to ethylene until day 4 (Figure 2). These results indicate that changes in these gene transcripts in response to ethylene are slow.

### 3.3 Ethylene alters lectin binding to cells

To follow up on the transcriptional changes we observed, we sought to determine to what extent these transcriptional changes correlate with biochemical changes. We previously discovered that Etr1 signaling affects extracellular components such as type IV pili to alter phototaxis and perhaps other physiological responses (Lacey & Binder, 2016). EPS contains numerous sugar moieties that help the bacterium adapt to environmental changes (Kehr & Dittmann, 2015) and has been implicated in phototaxis, spontaneous cell sedimentation, and biofilm formation in *Synechocystis* (Burriesci & Bhaya, 2008; Fisher et al., 2013; Jittawuttipoka et al., 2013). Because ethylene affects all three of these physiological responses (Lacey & Binder, 2016) and caused the downregulation of cell envelope genes and a large number of gene transcripts annotated as glycosyltransferases, including genes previously implicated in EPS formation, we hypothesized that ethylene alters EPS production leading to physiological changes.

**Figure 3:**
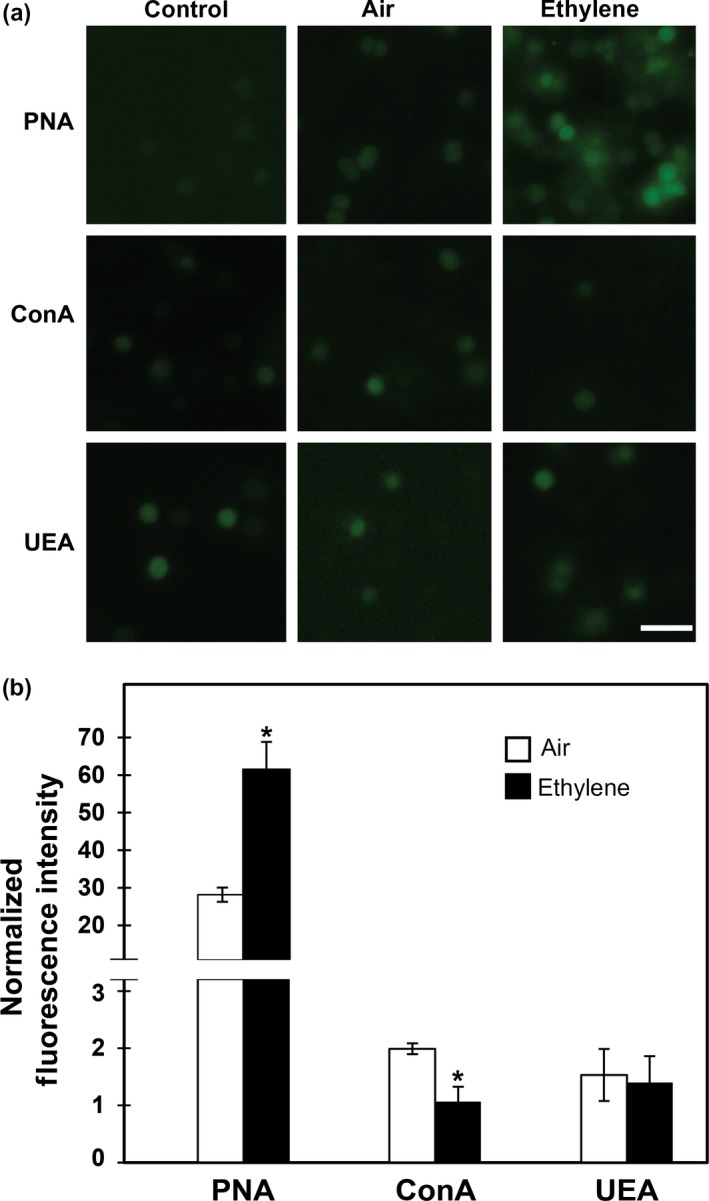
Lectin Binding to *Synechocystis* cells. Colonies of cells were kept in phototaxing conditions for 4d in the presence or absence of 1 μl/L ethylene. Cells were then labeled with FITC‐conjugated peanut agglutinin (PNA), concanavalin A (ConA) or *Ulex europaeus* agglutinin (UEA), observed with a fluorescence microscope, and images were acquired. Unlabeled cells were used as a control. (a) Fluorescence micrographs of *Synechocystis* cells. Images of cells in the absence of added lectin are shown as background controls. Scale bar = 10 μm. (b**)** Quantification of fluorescence intensity from FITC‐conjugated lectins bound to cells. Data have been normalized to fluorescence intensity in nonlabeled cells maintained in air and represent the average ± *SD*. *Statistically significant difference (*p *<* *.05) caused by application of ethylene as determined by Student's *t* test

We thus wished to know whether specific sugars on the cell surface were altered by ethylene treatment under these conditions. To test this, we examined lectin binding to the surface of *Synechocystis* cells using FITC‐labeled peanut agglutinin (PNA) that binds to galactose(β1‐3)*N*‐acetyl‐*D*‐galactosamine, concanavalin A (ConA) that binds to α‐D‐mannose and α‐D‐glucose, or *Ulex europaeus* agglutinin (UEA) that binds to α‐L‐fucose and N,N’‐diacetylchitobiose. The levels of background fluorescence in the absence of added lectin did not change significantly upon application of ethylene. When cells were kept in ethylene‐free air, PNA, ConA, and UEA labeled the surface of cells, resulting in fluorescence levels approximately 28‐fold, twofold, and 1.5‐fold, respectively, above the nonlectin controls (*p *<* *.05) (Figure 3). Application of 1 μl/L ethylene caused approximately a twofold increase in PNA binding and a decrease in ConA binding to background levels. Ethylene caused no measurable change in UEA binding levels. These results are consistent with a model where ethylene alters the composition of EPS to affect phototaxis, spontaneous cell sedimentation, and biofilm formation.

### 3.4 Real‐time RT–qPCR of protein‐encoding genes near *etr1*

**Figure 4:**
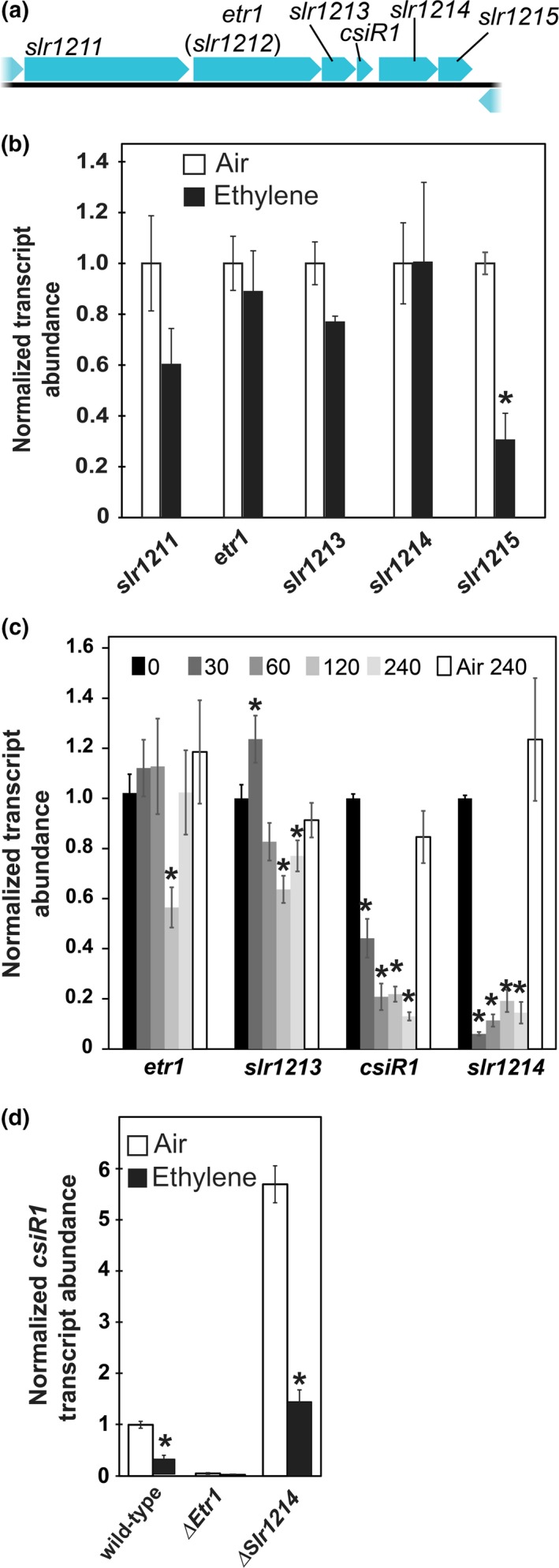
Real‐time RT–qPCR of Selected Transcripts Near *etr1* in the Genome. (a) Genomic structure around *etr1*. (b–d) Normalized transcript abundance of genes surrounding *etr1*. For panels b and d, cells were treated as described in Figure 1. For panel c, cells were maintained in phototaxis conditions for 1d in the absence of ethylene. At this time, the cells were treated with 1 μl/L ethylene for the times indicated. An air control at 240 min was also used. In all cases, RNA was isolated, and real‐time RT–qPCR was conducted. Data were normalized to the *trpA* reference gene transcript and then normalized to levels in wild‐type cells maintained in ethylene‐free air. For panel c, the data were normalized to the data from the 0 min time‐point. Data represent the average ± *SEM* of two‐five biological replicates with three technical replicates per biological replicate. In panels b and d, * designates statistically significant difference (*p *<* *.05) caused by application of ethylene as determined by Student's *t* test. In panel d, * indicates ethylene caused a statistically significant change (*p *<* *.05) from the levels at t = 0 as determined using two‐way ANOVA and Tukey's multiple comparisons test

The Etr1 receptor is modeled to signal via Slr1213 and Slr1214 which are encoded by genes near *etr1* on the genome (Figure 4a). RNA‐seq revealed that long‐term treatment with ethylene caused no significant changes in the transcript levels of *etr1*,* slr1213*, or *slr1214* (Table S1). We confirmed this using real‐time RT–qPCR on cells exposed to phototaxis conditions for 4 days (Figure 4b). We also looked at several other genes in this region of the genome and found that ethylene caused a statistically significant (*p *<* *.05) decrease in the transcript levels of *slr1215* (Figure 4b) which is consistent with our RNA‐seq data (Table 2). The transcript levels of *slr1211* were also reduced by ethylene, but the change was below the statistical cutoff used (*p *=* *.08). A similar decrease that was below the statistical cutoff was observed for this gene transcript in the RNA‐seq data (*q *=* *0.09) (Table S1).

The above results indicate that the genes coding for proteins that are posited to be part of the primary signaling pathway (*etr1*,* slr1213*,* slr1214*) are not regulated transcriptionally by long‐term ethylene treatment. However, it is possible that these genes are transiently affected by ethylene. Therefore, we examined the levels of these transcripts within the first 4 hr of ethylene exposure to ensure we were not missing transient changes. To do this, cells were maintained in phototaxis conditions for 1 day in ethylene‐free air, at which time they were treated with 1 μl/L ethylene for 0, 30, 60, 120, or 240 min. Air controls at 240 min were also examined. This showed that *etr1* has a small and transient decrease at 120 min and *slr1213* may have a small biphasic response where it first increases and then transiently decreases (Figure 4c). Thus, both of these genes have small, rapid changes to the application of ethylene that are transient so that by 4 days, there is no apparent effect of ethylene on these gene transcripts. By contrast, *slr1214* transcript levels were decreased over 10‐fold after 30 min of ethylene treatment and remained low by 240 min. This suggests that the return of *slr1214* transcript to pretreatment levels by 4 days is slower than for *etr1* and *slr1213*.

### 3.5 Ethylene affects transcript levels of *csiR1*

Small, noncoding RNAs have been shown to function at the transcriptional and posttranscriptional levels in *Synechocystis* (Georg et al., 2009; Hernández‐Prieto et al., 2012). Absorption of photons by Etr1 has been proposed to activate Slr1213 by phosphotransfer to a conserved Asp residue on Slr1213. Slr1213, in turn, is proposed to bind to the promoter of the small RNA, *csiR1* (Ramakrishnan & Tabor, 2016), which is located in the intergenic region between *slr1213* and *slr1214* (Figure 4a) (Kopf et al., 2014). We were consequently curious to know whether ethylene affects the levels of *csiR1*. However, small RNAs such as *csiR1* are not yet annotated in the genome annotation file used for RNA‐seq. Therefore, we examined the transcript abundance of *csiR1* using real‐time RT–qPCR and found that under phototaxing conditions (unidirectional white light), a four‐day treatment with ethylene causes approximately a threefold decrease in *csiR1* transcript abundance (Figure 4d). By contrast, exposure to UV light (without application of ethylene) causes an increase in *csiR1* transcript levels, suggesting ethylene and photons function antagonistically (Ramakrishnan & Tabor, 2016). As *csiR1* is likely to be part of the primary signaling pathway, we were curious to know how rapidly it responds to the application of ethylene. We found that a statistically significant decrease in *csiR1* transcript occurred within 30 min of application of ethylene and an apparent steady state was reached by 60 min after application of ethylene (Figure 4d). Thus, unlike the other genes proposed to be involved in signaling from Etr1, ethylene causes a rapid and prolonged decrease in *csiR1* transcript levels.

To determine whether the change in *csiR1* transcript depends on Etr1 signaling, we examined *csiR1* transcript abundance in cells with Etr1 deleted (ΔEtr1). The *csiR1* transcript levels in ΔEtr1 cells kept in ethylene‐free air were approximately 5% of the levels in wild‐type cells, and ethylene had little or no effect on *csiR1* transcript levels in this mutant (Figure 4d). These data support a model where signaling from Etr1 leads to increased *csiR1* levels, and ethylene inhibits this function causing a reduction in *csiR1* abundance. Ethylene is proposed to inhibit plant ethylene receptors and Etr1 (Hall, Chen, Findell, Schaller, & Bleecker, 1999; Lacey & Binder, 2016). The above results are consistent with this model and suggest a conservation of function between the ethylene receptors of *Synechocystis* and plants.

### 3.6 Slr1214 affects *csiR1* abundance

Slr1214 is proposed as a possible output of this signaling system and is required for signaling from Etr1 to affect phototaxis; current models for Slr1214 function posit that it acts downstream of Slr1213 and *csiR1* (Lacey & Binder, 2016; Narikawa et al., 2011; Ramakrishnan & Tabor, 2016; Song et al., 2011). However, the Slr1214 protein may also physically interact with the Etr1 receptor (Sato et al., 2007). Thus, Slr1214 may act as an alternative signaling pathway or provide feedback on Etr1. We therefore examined *csiR1* transcript levels in cells with Slr1214 deleted (ΔSlr1214). We previously showed that ethylene fails to alter the phototaxis of ΔSlr1214 cells (Lacey & Binder, 2016). Interestingly, *csiR1* transcript levels increased over fivefold in air in ΔSlr1214 cells compared to wild‐type cells (Figure 4d). Application of ethylene caused a reduction in *csiR1* transcript levels in ΔSlr1214 cells, suggesting that signaling via Slr1214 is not required for the reduction in *csiR1* by ethylene. These data are consistent with a model where *csiR1* is upstream of Slr1214 and slr1214 functions in negative feedback on this pathway. However, it is possible that deletion of Slr1214 had other effects such as simply stabilizing *csiR1* transcript resulting in higher levels.

## 4 DISCUSSION

Ethylene receptors have been highly studied in plants and models developed for ethylene‐binding and signal transduction (Lacey & Binder, 2014; Shakeel, Wang, Binder, & Schaller, 2013). By contrast, very little is known about ethylene receptors in nonplant species and only one ethylene receptor has been characterized in a nonplant (Henry et al., 2017; Lacey & Binder, 2016; Rodriguez et al., 1999). In these prior studies, we demonstrated that *Synechocystis* contains a functional ethylene receptor that affects physiological processes such as phototaxis and biofilm formation and biochemical processes such as formation of type IV pili and levels of photosystem II. Other laboratories have shown that Etr1 also functions as a photoreceptor that is posited to signal to Slr1213 via phosphorelay. The phosphorylated Slr1213 is predicted to increase the transcription of the noncoding, small RNA, *csiR1*, and *slr1214* (Narikawa et al., 2011; Ramakrishnan & Tabor, 2016; Song et al., 2011; Ulijasz et al., 2009).

To gain a better understanding about ethylene signal transduction in *Synechocystis*, we examined global transcript changes in response to long‐term ethylene treatment using RNA‐seq. We found that the transcription of over 500 genes was altered in various functional categories and the transcription of 63 genes altered twofold or more. Global transcriptomic studies have been carried out on *Synechocystis* using either microarrays or RNA‐seq to examine the effects of various conditions (Anfelt, Hallström, Nielsen, Uhlén, & Hudson, 2013; Hernández‐Prieto et al., 2012; Hihara, Kamei, Kanehisa, Kaplan, & Ikeuchi, 2001; Huang, McCluskey, Ni, & Larossa, 2002; Kizawa, Kawahara, Takimura, Nishiyama, & Hihara, 2016; Klähn et al., 2015; Kopf et al., 2014; Lau, Foong, Kurihara, Sudesh, & Matsui, 2014; Wang, Postier, & Burnap, 2004; Zhang et al., 2008). Even though there is some overlap in the gene transcripts that are changed in these studies compared to this study, the overall pattern of transcriptional changes that occurs with ethylene does not match what occurs in these other studies, indicating that ethylene is having unique effects on gene transcript levels in *Synechocystis* cells.

Interestingly, while this article was under review, an article was published where *Synechocystis* was bioengineered to produce high levels of ethylene (Kuchmina et al., 2017). This study used microarray and Northern blot analyses to examine transcriptional changes. Consistent with our study, they observed a decrease in *csiR1* and *slr1214* transcripts 4 hr after initiating expression of an enzyme that leads to ethylene production. Additionally, levels of *csiR1* remained low for up to 48 hr. However, other than these two genes, there was very little overlap between the genes we identified and those identified by Kuchmina et al. (2017). It should be noted that there are numerous differences in the conditions used in these two studies. In particular, our samples were on agar plates and exposed to unidirectional light for 4 days in ethylene‐free air or 1 μl/L exogenously applied ethylene. By contrast, the samples used by Kuchmina et al. (2017) were grown in liquid culture and grown with induction for 4 hr to increase the abundance of ethylene‐forming enzyme, resulting in ethylene levels of 1500 μl/L ethylene. It is likely that the differences between our transcript results and theirs are due to these large differences in the conditions used.

Small RNAs have been shown to function at the transcriptional and posttranscriptional levels in *Synechocystis* (Georg et al., 2009; Hernández‐Prieto et al., 2012). In response to light, Slr1213 is proposed to bind to the promoter of *csiR1*, which is located in the intergenic region between *slr1213* and *slr1214*, causing an increase in *csiR1* levels (Ramakrishnan & Tabor, 2016). We found that long‐term treatment with ethylene leads to a reduction in *csiR1* transcript abundance, showing that it is likely to be involved in ethylene signaling and that ethylene acts oppositely to light. The transcription of *csiR1* is also downregulated in conditions of high inorganic carbon such has high CO_2_ levels (Klähn et al., 2015; Kopf et al., 2014; Wang et al., 2004). However, except for the downregulation of *csiR1* by both ethylene and high inorganic carbon, there is little, if any, overlap between the gene transcripts regulated by high carbon conditions and application of ethylene.

We also examined the time‐course of changes in the protein coding genes near *etr1* on the genome as well as *csiR1*. This showed ethylene causes a small rapid and transient change in *etr1* and *slr1213*. Ethylene caused a larger decrease in the transcript levels of *slr1214*. By 4d of ethylene treatment, there was no significant difference in the transcript levels of *etr1*,* slr1213*, or *slr1214* in air versus ethylene; this is similar to the results found with RNA‐seq. Thus, the effects of ethylene on the transcript abundance of these three genes are transient. Application of ethylene also caused a rapid decrease *csiR1*. However, unlike *etr1*,* slr1213*, and *slr1214*, the transcript levels of *csiR1* remained low up to 4d of ethylene treatment. This is interesting because it has been suggested the *csiR1* and *slr1214* are cotranscribed from a common start site for transcription, leading to the expectation that their transcripts would have similar patterns of change (Klähn et al., 2015; Kopf et al., 2014; Mitschke et al., 2011; Ramakrishnan & Tabor, 2016). At least part of this difference between the levels of *csiR1* and *slr1214* may be due to a transcriptional termination site between the two (Klähn et al., 2015; Kopf et al., 2014). It is also possible that the transcripts of *csiR1* and *slr1214* are regulated differently so that over time, *slr1214* levels increase due to decreases in the rate of degradation, but *csiR1* levels do not. The rapid changes observed for these genes are different for what we observed for *sll5043*,* slr1452*, and *pilB1* that respond more slowly to ethylene. This is consistent with a model where *sll5043*,* slr1452*, and *pilB1* are part of downstream pathways that mediate physiological changes, rather than part of the primary signaling pathway.

**Figure 5:**
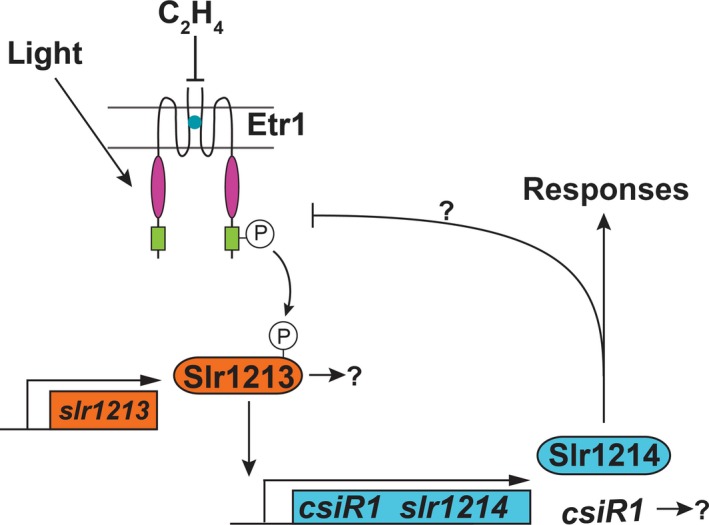
Model for ethylene and light signaling via Etr1. In this model, light stimulates Etr1 to autophosphorylate on a His residue. This is then transferred to a conserved Asp on Slr1213 that acts as a transcriptional activator of *csiR1* and *slr1214*. Slr1214 is modeled to be the required output of the pathway. However, it is also possible that Slr1213 and *csiR1* have output functions too. Our data indicate that ethylene acts antagonistically to light. Deletion of Slr1214 resulted in an increase in *csiR1* levels, indicating there may be negative feedback from Slr1214. However, it is also possible that deletion of Slr1214 resulted in increased stability of *csiR1*. Based on results from this study and prior reports (Lacey & Binder, 2016; Narikawa et al., 2011; Ramakrishnan & Tabor, 2016; Song et al., 2011)

A linear pathway has been proposed previously where, upon stimulation by light, Etr1 signals to Slr1213 via phosphorelay (Figure 5). Phosphorylated Slr1213 in turn binds to the promoter of *csiR1‐slr1214,* increasing the transcription of *csiR1* and *slr1214* to affect downstream signaling (Lacey & Binder, 2016; Narikawa et al., 2011; Ramakrishnan & Tabor, 2016; Song et al., 2011). Slr1214 is required for signaling from Etr1 (Lacey & Binder, 2016; Narikawa et al., 2011; Song et al., 2011). There are several possible mechanisms by which deletion of Slr1214 affects *csiR1* transcript levels. One is that Slr1214 provides feedback inhibition on the system so that when it is removed, *csiR1* levels increase (Figure 5). However, we cannot rule out other possibilities such as that deletion of Slr1214 may simply cause stabilization of the *csiR1* transcript, leading to elevated levels.

Slr1214 seems to be required for output from this pathway (Lacey & Binder, 2016; Narikawa et al., 2011; Song et al., 2011). However, we cannot rule out that there are also Slr1214‐independent pathways that occur from Slr1213 or *csiR1* or both. For instance, signaling from Slr1213 could involve additional points of transcriptional activation, while *csiR1* could be regulating translation or protein function involved in responses to ethylene (Georg et al., 2009; Hernández‐Prieto et al., 2012; Ramakrishnan & Tabor, 2016). In support of a role for a small RNA‐like *csiR1* affecting motility is the observation that the RNA chaperone HFq affects *Synechocystis* motility and type IV pilus function (Dienst et al., 2008; Schuergers et al., 2014). More complex scenarios are also possible. For instance, a number of small, noncoding RNAs have been identified that both accumulate transcript and act as a 5′ UTR for a downstream gene; these have been termed “actuatons” (Kopf, Klähn, Scholz, Hess, & Voß, 2015). *CsiR1* is one of the small RNAs identified as an actuaton where it both changes transcript abundance and acts as the 5′UTR for *slr1214*. Thus, *csiR1* could have multiple functions in this signaling pathway. It is also likely that Slr1213, *csiR1*, and Slr1214 provide points of signal integration with other environmental signals as iron starvation causes a reduction in the transcript levels of *slr1213* and *slr1214* (Hernández‐Prieto et al., 2012) and low‐carbon conditions upregulate *csiR1* and *slr1214* transcripts (Klähn et al., 2015; Langmead et al., 2009; Wang et al., 2004). These results suggest that iron and carbon availability may affect signaling from Etr1; however, the significance of such signaling is still unclear. The regulation of *csiR1* by Slr1214 that we observed provides another mechanism by which iron and carbon availability could modulate the Etr1 signal transduction pathway.

We wished to know whether or not the changes in gene transcripts were reflected in biochemical changes. We have previously found that Etr1 signaling affects extracellular components such as type IV pili and possibly EPS (Lacey & Binder, 2016), and it is known that both type IV pili and EPS affect phototaxis, biofilm formation, and spontaneous cell sedimentation of *Synechocystis* cells (Bhaya, Bianco, Bryant, & Grossman, 2000; Bhaya, Watanabe, Ogawa, & Grossman, 1999; Burriesci & Bhaya, 2008; Fisher et al., 2013; Jittawuttipoka et al., 2013). Cell envelope genes, as well as several other genes linked to EPS formation, were affected by ethylene treatment, correlating with the changes in lectin binding to *Synechocystis* cells. This supports a model where one effect of ethylene is to alter the composition of EPS, leading to alterations in physiology. The exact nature of these changes has yet to be resolved, but our results show that there is not simply a global increase or decrease in EPS. Rather, ethylene is having different effects on specific carbohydrates on the cell surface as ethylene caused a decrease in ConA binding and an increase in PNA binding, indicating a decrease in either α‐D‐mannose or α‐D‐glucose or both and an increase in galactose(β1‐3)*N*‐acetyl‐*D*‐galactosamine. We also observed changes in the abundance of several pilin‐related gene transcripts correlating with our previous observation that ethylene alters type IV pili (Lacey & Binder, 2016). Together, these data provide support for a model where ethylene signaling in *Synechocystis* alters the physiology of the cells by regulating extracellular components.

The transcript levels of two genes that encode for phycobilisome degradation enzymes are reduced, suggesting that the light harvesting complex for photosynthesis is also altered by ethylene. Phycobilisomes can potentially act as antenna complexes for both photosystem I and II in *Synechocystis* where they can contribute to energy dissipation under high‐light conditions (Kondo, Ochiai, Katayama, & Ikeuchi, 2007). Ethylene is produced when dissolved organic compounds are exposed to light. Thus, it is possible that environmental ethylene is functioning, in part, to facilitate adaptive responses to high‐light conditions. As such, it could be considered a stress signal, similar to its function in plants. The fact that EPS production in *Synechocystis* changes and cells form biofilm in response to stress is compatible with this idea (Jittawuttipoka et al., 2013; Song, Zhao, Zhang, Mu, & Pan, 2016). However, this seems unlikely as there is little overlap in the transcriptional changes caused by high light (Huang et al., 2002) compared to ethylene.

In summary, ethylene regulates the transcription of many genes, causing alterations in both intra‐ and extracellular processes. We propose that these changes lead to a variety of physiological changes including motility, biofilm formation, and cell sedimentation and point to the importance of ethylene as an environmental cue that alters cyanobacteria behavior. A model has been proposed where phototaxis signaling pathways affect either orientation of movement or cell surface components involved in motility such as EPS and type IV pili (Schuergers et al., 2017). Pathways involved in orientation of movement would likely involve the relocalization of at least some components of the pathway in response to directional light. By contrast, pathways involved in cell surface modifications to alter motility would not necessarily show changes in localization. Our data do not address whether or not the Etr1 pathway is involved in orientation of movement. However, our data do support the idea that this pathway affects important components involved in motility. It remains to be determined whether or not this pathway is also involved in the regulation of the direction of movement.

The ecophysiological role of ethylene for *Synechocystis* remains an open question. Because ethylene is produced when sunlight interacts with organics dissolved in water (Ratte et al., 1993, 1998; Wilson et al., 1970), it is possible that ethylene is acting as a diffusible signal to enhance responses to light or as a cue to help the cells find optimal light conditions. Alternatively, *Synechocystis* may use ethylene emitted from another organism as a cue to form a symbiotic interaction with that organism (Schuergers et al., 2017). Ethylene receptors are predicted to also be present in other bacterial species as well as several fungi (Bakshi & Binder, 2018; Hérivaux et al., 2017). Therefore, it is likely that ethylene is altering the physiology of diverse microorganisms. The nature of these alterations in these species remains to be determined.

## AUTHOR CONTRIBUTIONS

R.F.L., C.J.A., and A.B. performed the experiments. R.F.L. and B.M.B. designed experiments and wrote the manuscript with contributions from C.J.A. and A.B. B.M.B. supervised the research.
