# Supplementary material for: Ethylene causes transcriptomic changes in Synechocystis during phototaxis
Source: Plant Direct. 2018 Mar 15;2(3):e00048. doi: 10.1002/pld3.48 (PMC6508509; doi:10.1002/pld3.48)
Supplement: Supplementary file 1 [file PLD3-2-e00048-s001.pdf]

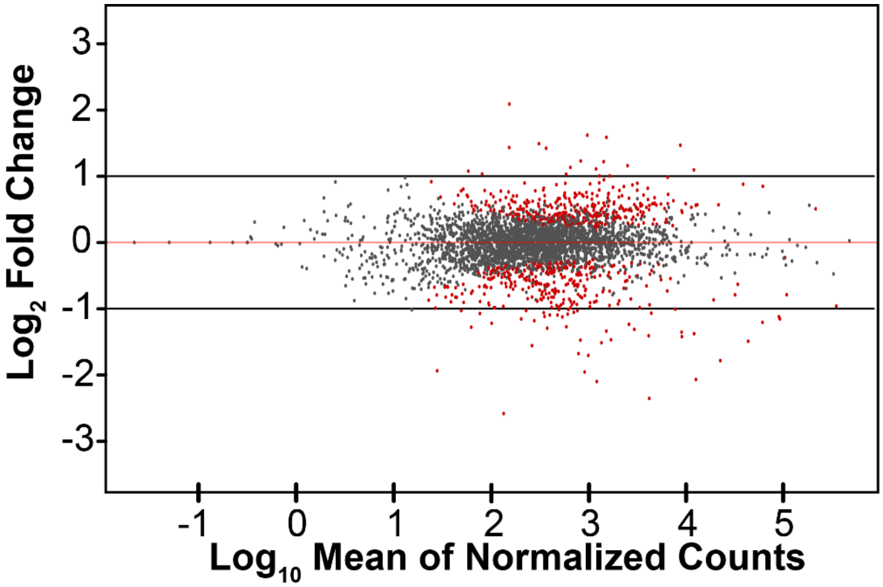

Supplemental Figure 1. MA Plot Showing Global Gene Transcript Changes Caused by the Application of Ethylene to *Synechocystis* Cells. Cells were maintained in phototaxis conditions for 4d in air or 1 µL L^{-1} ethylene. RNA-seq and analysis was carried out as described in the materials and methods and differential gene expression determined. Red indicates gene transcripts that are differentially expressed upon treatment with ethylene with a false discovery rate < 0.05. Horizontal black lines indicate a 2-fold change.

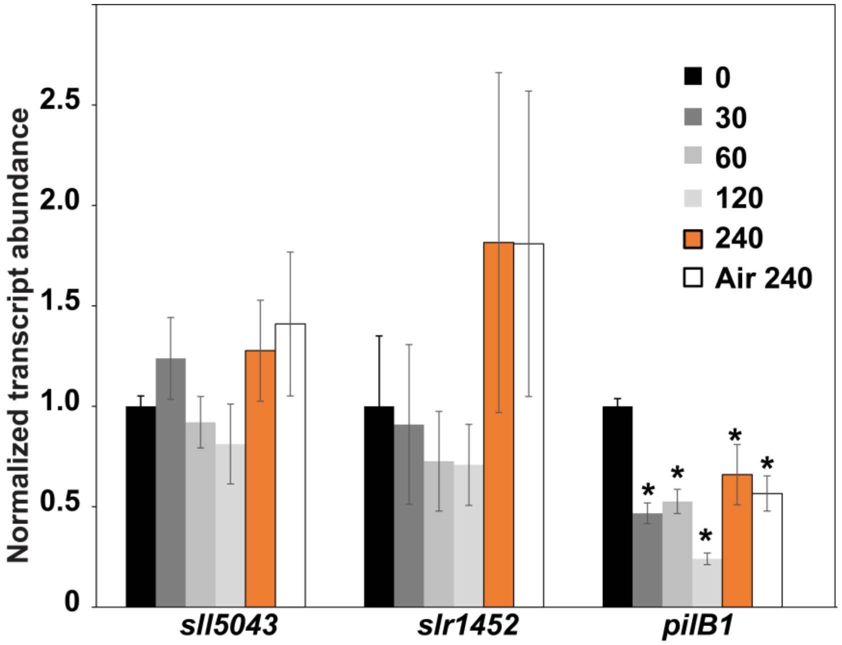

Supplemental Figure 2. Real-time RT-qPCR of Selected Genes at Early Time-points After Ethylene Treatment. Cells were maintained in phototaxis conditions for 1d in the absence of ethylene. At this time the cells were treated with 1 µL L^{-1} ethylene for the times indicated. An air control at 240 min was also used. In all cases, RNA was isolated and real-time RT-qPCR conducted. Data were normalized to the *trpA* reference gene transcript, then normalized to levels in the 0 min time-point. Data represents the average ± SEM of 2 biological replicates with 3 technical replicates per biological replicate. * indicates a statistically significant change ($P < 0.05$) from the levels at t=0 as determined using two-way ANOVA and Tukey's multiple comparisons test.
