# Supplementary material for: Ethylene causes transcriptomic changes in Synechocystis during phototaxis
Source: Plant Direct. 2018 Mar 15;2(3):e00048. doi: 10.1002/pld3.48 (PMC6508509; doi:10.1002/pld3.48)
Supplement: Supplementary file 3 [file PLD3-2-e00048-s003.pdf]

Responses to Handling editor/ Reviewer #1 (our responses in bold):
Additionally, the authors should compare their data with the recently published article by Kuchmina et al. (2017) in the journal Microbiology ("Ethylene production in Synechocystis sp. PCC 6803 promotes phototactic movement") and mention in which way the present paper advances knowledge on the topic.

The paper by Kuchimina *et al*. came out during review of our manuscript. We have now included information from this paper and cite it in the revision of our manuscript. Specifically: a) we have added a sentence to our introduction (lines 67-68) about how they see enhanced phototaxis in their ethylene overproducing cells in agreement with our prior research; b) we've expanded the discussion regarding the differences in their results versus ours (lines 310-321). Essentially, there is virtually no overlap in the genes they identify compared to our data; the exceptions are *csiR1* and *slr1214*. However, their conditions were drastically different from those used in our study. This is actually something they point out in their discussion when comparing their results with our prior paper examining the physiological responses to ethylene. In particular, we used phototaxis conditions (unidirectional light) on agar plates with a treatment of 1 ppm ethylene delivered exogenously for 4d. Their samples were grown in liquid culture with agitation (so the light was not unidirectional) and a concentration of ethylene of 1500 ppm after 4h of induction to increase ethylene production. Most responses in plants saturate by 1 ppm so this is a very high level of ethylene from a biological standpoint. We have never treated Synechocystis cells with such a high level of ethylene, however, we have unpublished results showing that when we gas liquid cultures with 100 ppm ethylene they start to become yellow suggesting such high levels of ethylene over several days is harmful to the bacteria. Given the drastic differences in conditions, especially the very high ethylene levels used by Kuchimina et al, it's not surprising that there is hardly any overlap between our transcript results and theirs.

Due to the fact that there are some overlaps with this new paper, I consider that some additional RT-qPCR of new genes (3-4 selected among transporters, glycosyltransferases, transcription factors, genes coding for enzymes involved in carbohydrate metabolism) at early time-points after ethylene treatment could bring additional novel information. In fact, RNAseq was performed 4d following ethylene treatment and important information may have been missed. This was actually one comment provided by reviewer 1 that failed to send the report on time.

As pointed out above, there is virtually no overlap in the transcript analyses between these papers. However, based on this you (or reviewer 1, we cannot tell) suggest that several genes be examined at earlier time-points. We already did this in the last revision examining changes as early as 30 min after the addition of ethylene. We don't feel that analysis of a few additional genes will increase our understanding. We hope to do RNA-seq analyses at these earlier time-points to gain a global perspective of changes in transcription over time, but this is beyond the scope of the current submission.

Responses to Reviewer #3 (our responses in bold):
The paper by Lacey et al. analyzes changes in gene expression in response to external application of ethylene. In a recent paper this group showed that ethylene has effects on

phototaxis and several other physiological characteristics of Synechocystis 6803 cells. Now they have used RNAseq to determine changes in transcript accumulation and several transcript changes have been also verified by RT-PCR analysis. I think this is interesting information which shed light on the effect of this important plant hormone on cyanobacteria.

This reviewer feels that the authors responded adequately to the comments of the previous reviewers. I do not think that additional experimentation is needed because the signal transduction mechanism in the ethylene-responsive two-component system is comparably well understood already. I just have some minor new comments regarding interpretation of the data. I think that the authors should be more careful with some of the conclusions.

1. line 35-37 (line 244, line 251, line 280): The increase in csiR1 transcript accumulation in a Δslr1214 strain could be also explained by a stabilization of the csiR1 transcript by deletion of the downstream gene. In addition, a resistance cassette was inserted into this region and it was not clear for me in which orientation this was done. In general, in my opinion, Ramakrishnan and Tabor (2016) showed that csiR1 and slr1214 are co-transcribed and that this putative ncRNA is most probably just the 5`UTR of the slr1214 mRNA. Of course, it is still possible that the 5`UTR (or csiR1) has some own functions, but it is also clear that the ETR1-Slr1213 signalling targets the csiR1-slr1214 promoter. I think this should be explained better. Differences between csiR1 and slr1214 transcript accumulation can be easily described by different transcript stabilities and RNA processing and most probably have nothing to do with a putative feedback mechanism (in my opinion). However, you might also discuss this complex scenario in line with the new actuaton concept (Kopf and Hess, 2015) as CsiR1 is discussed by these authors as an example of an actuaton.
We thank the reviewer for suggesting these ideas. In response, we have removed the idea of feedback from the abstract and introduction. We did not change lines 244, 251, or 280 (as numbered in the original manuscript) since these were simply reporting the observations. However, based on the reviewers comments we have changed lines 286-287 of the results and incorporated many of the ideas suggested into the discussion (lines 343-345; 353-357; 365-369). We assume the reviewer meant the paper by Kopf et al , 2015 *Sci. Reports*; this paper has been used and cited for some of the revisions. These suggestions have resulted in re-writing parts of the discussion. Even though we agree with the ideas put forth by the reviewer, at this time we still cannot rule out feedback by Slr1214. We certainly hope to test this model in the future. However, because of this uncertainty, we have slightly modified our model (fig 5) to have a question mark on the part indicating feedback. The legend has been altered accordingly.

2. Please be a bit more careful with annotation of genes
line 151 and table 1: Slr0551 is the Synechocystis RNaseJ homolog, but it is not part of the degradosome, because no degradosome has been described in cyanobacteria so far.
Removed from text and changed in table 1.

Line 163-164: these two open reading frames are nblA1 and nblA2 and these are not phycobilisome degradation enzymes. These are small adapter proteins (but no enzymes) which are involved in phycobilisome degradation.

Reworded on these lines and in the introduction.

Table 2: slr0079 is actually the pilB2 homolog. I think this would be informative for the reader

This information has been added to table 2.

For these 3 comments, we thank the reviewer for pointing these issues out. We had simply followed the annotation provided on-line, but given this reviewers comments, these parts of the manuscript have either been removed or re-worded.

3. I am not sure when the authors submitted this manuscript initially. But meanwhile there was a paper by Kuchmina et al. (2017) which describes transcript changes in response to ethylene production by the cells. I think this should be cited and discussed somewhere.
We are glad the reviewer pointed this out. We saw this too. This paper came out while our manuscript was under review. We have added a sentence about this paper in our introduction (lines 67-68) and added some writing to the discussion regarding the similarities and differences in their results versus ours (lines 310-321). Essentially, there is very little overlap in the genes they identify compared to our data. However, their conditions were drastically different from those used in our study. In particular, we used phototaxis conditions on agar plates with a treatment of 1 ppm ethylene delivered exogenously for 4d. Their samples were grown in liquid culture without directional light and a concentration of ethylene of 1500 ppm after 4h of induction to increase ethylene production. Most responses to ethylene in plants saturate by 1 ppm. We have never treated Synechocystis cells with such a high level of ethylene, however, we have unpublished results showing that when we gas liquid cultures with 100 ppm ethylene they start to become yellow suggesting such high levels of ethylene over several days is harmful to the bacteria. While not reported in our manuscript, we find that even 1ppm is supersaturating for the physiological responses we examine and we recently determined that much lower levels of ethylene elicit a response similar to 1ppm. We are now determining the lowest effective concentration to elicit a response and hope to have this as part of a future study.

In addition, I have some minor points.
Line 132: correlate (grammar) **Fixed**
Line 137: Synechocystis: italic **Fixed**
Line 152: sll1598 interacts with sll11598?? this is a mistake, obviously **Fixed. Was supposed to be sll1599.**
Line 257: which light (quality)? **Reworded.**
Line 393: Is there any citation for the statement that movement of Synechocystis is faster in mats? To my knowledge, Synechocystis movement has never been investigated in

mats or biofilms. **The reviewer is correct and we have modified this sentence to better reflect what we meant to say (lines 418-420).**

Line 408: I did not find any information in the Lacey and Binder (2016) paper about the construction Δslr1214 strain – **We used the methods of Song et al (2011) to generate this deletion; this is cited in Lacey and Binder (2016). Since this could lead to confusion, we have added the Song et al citation where we mention that this strain has been previously described (line 433).**

Table 2: it would be nice to inform the reader somewhere that all the slr50xx loci are on a plasmid. **We have added replicon information in both tables 1 and 2 (following the format used by Kuchmina et al) to address this suggestion. It didn't seem to make sense to only give this information for the slr50xx loci since several other loci are also on a plasmid.**

I think that Table3 and table 4 can be moved to the supplement
We would prefer to keep these tables in the main text.
